# Nursing students’ knowledge, attitude, self-efficacy in blended learning of cardiopulmonary resuscitation: a randomized controlled trial

**DOI:** 10.1186/s12909-019-1848-8

**Published:** 2019-11-09

**Authors:** Hyunjung Moon, Hye Sun Hyun

**Affiliations:** 1 College of Nursing, Incheon Catholic University, 12 Haesong-ro, Yeonsu-gu, Incheon, 22000 South Korea; 2 Department of Nursing, Sangmyung University, 31 Sangmyungdae-gil, Dongnam-gu, Cheonan, Chungnam 31066 South Korea

**Keywords:** Blended learning, Cardiopulmonary resuscitation, Nursing, Knowledge, Attitude, Self-efficacy

## Abstract

**Background:**

Although various forms of online education are on the rise worldwide, effects of such innovative approach are yet to be validated. This study analyzes whether blended learning cardiopulmonary resuscitation (CPR) education that integrates e-learning and face-to-face education is effective in improving nursing students’ knowledge, attitude, and self-efficacy.

**Methods:**

A randomized controlled design was used. The participants of this study were 120 nursing students randomly assigned to the intervention group (*n* = 60) or the control (*n* = 60). The intervention group was trained using a blended learning CPR education program. Self report questionnaires with knoweldge, attitude, and self-efficacy were all used in the pre and post intervention. Differences before and after the education of each group were analyzed with a paired *t*-test, and the differences between the two groups were analyzed with ANCOVA with knowledge as the covariate.

**Results:**

The findings indicated that the intervention group had significantly higher knowledge scores (intervention: 16.40 ± 1.56, control: 6.46 ± 2, *p* < .001), and emotional attitude (intervention: 40.85 ± 8.01, control: 36.05 ± 6.87, *p* = .002) about CPR than the control group, but other outcomes did not differ between groups.

**Conclusions:**

In this monocentric study, a blended learning CPR program that integrated videos and face to face lecture was found effective in improving nursing students’ knowledge and attitudes regarding CPR.

## Background

Heart disease, along with cancer, is one of the leading causes of death [1]. In fact, an estimated 31% of the deaths worldwide each year are due to cardiovascular diseases (CVDs), and cardiac arrest and stroke account for 80% of total CVD deaths. In such a context, it is very important for health workers to be prepared to administer CPR to patients with CVD [2]. Nurses are likely to be first responders, because they spend significant time alongside patients and are often the first to realize when a patient is experiencing an in-hospital cardiac arrest. Thus, it would be beneficial for nursing students to have proper knowledge and high self-efficacy about CPR to strengthen their skills for future use [3, 4]. A study that analyzed nursing students’ self-efficacy as an outcome of a simulation-based Basic Life Support education program [3] and another study both reported that nursing students who performed chest compression properly have higher self-efficacy [5].

To be able to respond during cardiac arrest situations promptly and effectively, nurses must be skilled at, prepared for, and updated on life-saving procedures [6], which may require repeated CPR training [7]. In particular, the attitudes of nurses and nursing students toward attempting CPR are an important factor in the prompt and successful responses to cardiac emergencies [8, 9]. Thus, it would be appropriate to include the first responder’s attitude toward CPR practice as a variable when exploring the factors that are related to CPR performance.

Recently, several studies on various education programs utilize e-learning with computers and internet. Blended learning enables self-directed, iterative learning, which may render it more effective than traditional training education in improving nursing competence [10]. A study that assessed the persistence of the effects of a CPR education based on theoretical lectures and training [11] found that knowledge and self-efficacy significantly decreased after 3 months, suggesting that continuous education and training are essential. CPR e-learning is effective for teaching knowledge, attitude, and technique [12–14], and it has been reported to be more effective than instructor-centered CPR education in improving knowledge, self-efficacy, and performance [15, 16]. However, some studies have reported that e-learning does not produce significantly better outcomes compared to traditional face-to-face education [17]. Further, applying both video training and theoretical education together was more effective than using video training alone, and it has been suggested that additional randomized controlled trials (RCTs) are needed to accurately assess the effects of e-learning [7, 18, 19].

Although various forms of online education are on the rise worldwide [18, 19], the effects of such innovative approaches have yet to be validated [18]. In South Korea, the Ministry of Health and Welfare and the Korea Centers for Disease Control and Prevention (KCDC) develop and distribute standard CPR e-learning materials with government funding, but no study has measured their effects. Thus, this study aims to examine whether a standardized CPR e-learning program, devised by a Korean public health institution in collaboration with experts, is effective.

## Methods

### Design

This study used a randomized controlled design. Written informed consent was obtained from all participants before inclusion in the study, which was previously approved by the Sangmyung University Institutional Review Board (SMUIRB AP-2017-003).

### Research hypotheses

Hypothesis 1. The intervention group who receive blended learning CPR education will have a higher CPR knowledge score after education than that before education.

Hypothesis 2. The intervention group who receive blended learning CPR education will have a higher CPR attitude score after education than that before education.

Hypothesis 3. The intervention group who receive blended learning CPR education will have a higher CPR self-efficacy score after education than that before education.

Hypothesis 4. The intervention group who receive blended learning CPR education will have a higher CPR knowledge score after education than that of the control group.

Hypothesis 5. The intervention group who receive blended learning CPR education will have a higher CPR attitude score after education than that of the control group.

Hypothesis 6. The intervention group who receive blended learning CPR education will have a higher CPR self-efficacy score after education than that of the control group.

### Participants

**Fig. 1:**
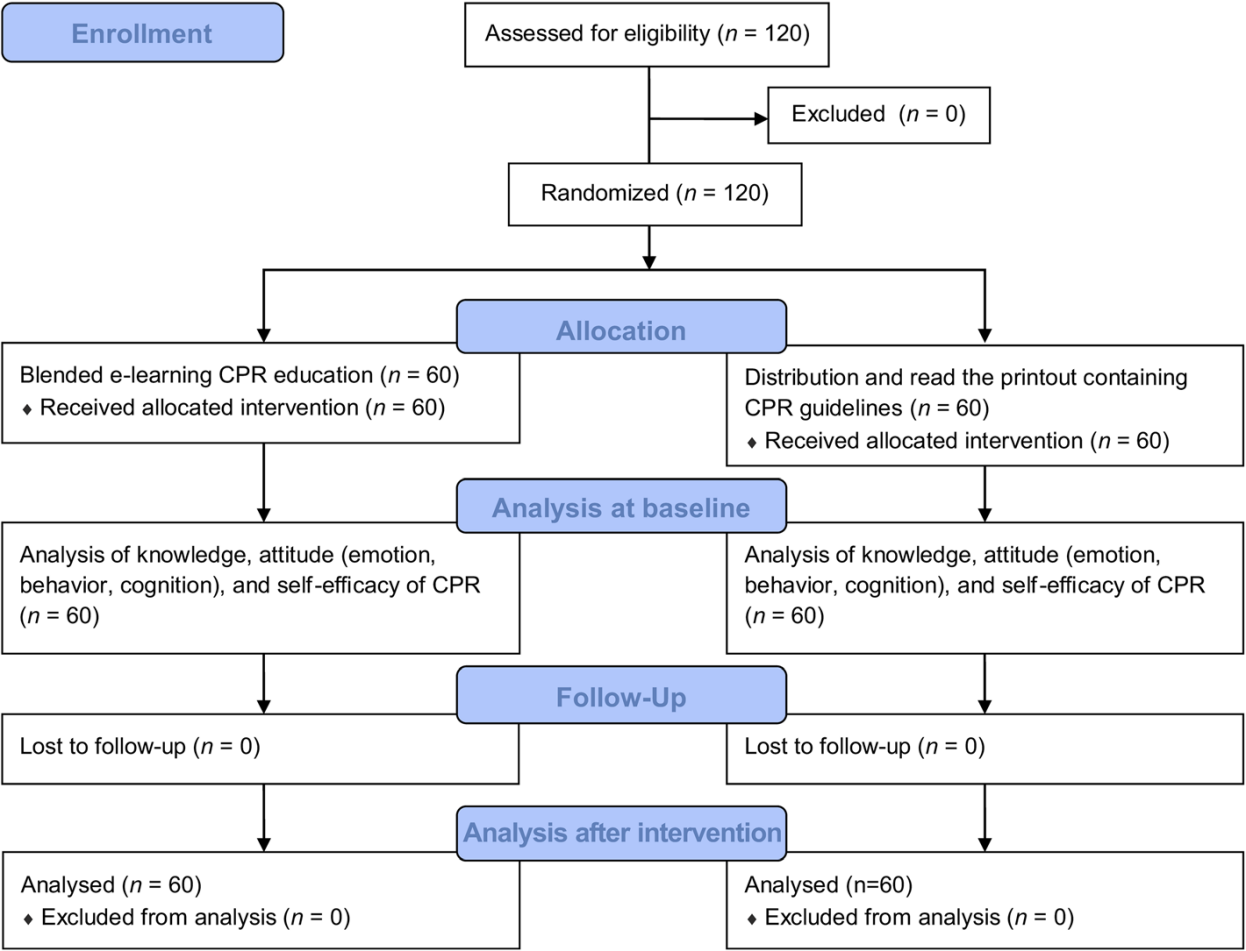
Flow diagram

This study adheres to the CONSORT guidelines and was a prospective randomized controlled trial aiming to identify effects of blended learning CPR education on nursing students’ CPR-related knowledge, attitude, and self-efficacy. The minimum sample size for analyzing differences between two groups with a two-tailed test was calculated using G*Power 3.1. With a statistical significance level of 0.05, power of 0.85, and effect size of 0.60, the sample size was calculated to be 51 for each group, totaling 102. Considering potential dropouts, 120 nursing students were recruited from a single institution. Nursing students who provided their written informed consent to participate in this study, had not completed the emergency department training course, and had never received blended learning CPR education were eligible to participate in the study. We excluded fourth-year students, as they had completed the emergency department training course, while 40 first-year, 40 s-year, and 40 third-year students were recruited. Education was administered and data collected between September and November 2017 (Fig. 1).

### Randomization

For each grade-level, students were assigned to the intervention and control groups based on the order of entering the lecture room, where odd numbers were assigned to the intervention and even numbers were assigned to the control group. To adjust for differences among years in school, twenty students from each grade-level were randomly assigned to the intervention and control groups each, resulting in a total of 60 students in the intervention group and 60 students in the control group.

### Intervention of blended learning CPR education

The blended learning CPR education program was designed as a four-session program. In Session 1, program orientation was given. In Session 2, students watched a video titled “How to perform chest compression CPR and use automated defibrillator”. The video described the definition of CPR, the overall CPR process, basics of chest compression CPR, and how to use an automated defibrillator. In Session 3, students watched a video titled “Basic course for standard CPR education program”. The video contained information about cardiac arrest cases and need for CPR, successful CPR cases, chest compression process, chest compression training, cases in which no one is available to help, how to use the speaker feature of a cell phone, repeated CPR training, how to use an automated defibrillator, precautions for using a defibrillator, how to use an emergency medical information application, and how to deliver rescue breaths. In Session 4, students were given a lecture using a printout made by the investigator based on the key contents of the KACPR guideline and 2010 AHA guideline for CPR and emergency cardiovascular treatment (Table 1). The printout containing CPR guidelines was also distributed to and read by the control group. In other words, the control group had only the 90-min lecture.

**Table 1:** Overview of blended learning CPR education program

Session (Time)	Themes	Content of Education
1 (60 min)	Introduction	▪ Introduction to the program & ice breaker ▪ Promotion participation & interaction ▪ Motivation
2 (30 min)	Outline of chest compression CPR and how to use an automated defibrillator (Produced by KACPR and managed by MOHW and KCDC)	▪ Definition of CPR ▪ Basic explanation of the overall CPR process ▪ Basic explanation of chest compression CPR ▪ Basic explanation of how to use automated defibrillator
3 (50 min)	Standard CPR education program (Produced by KACPR and managed by MOHW, KCDC, Ministry of Education, Ministry of Public Safety and Security)	▪ Examples of cardiac arrest and the need for CPR ▪ Examples of CPR rescue ▪ Chest compression resuscitation course ▪ Chest compression practice ▪ If there is no one who can help you around, the method of CPR ▪ How to use the speaker feature on a cell phone ▪ Repeated CPR training ▪ How to use an automated defibrillator ▪ Precautions when using an automated defibrillator ▪ How to use the emergency medical information application ▪ How to deliver rescue breaths
4 (90 min)	Lecture using a printout	▪ Basic concept of CPR ▪ CPR procedure ▪ Details of each step of CPR

*Note. CPR* Cardiopulmonary resuscitation, *KACPR* Korean Association of Cardiopulmonary Resuscitation, *MOHW* Ministry of Health and Welfare, *KCDC* Korea Centers for Disease Control and Prevention

The pre-intervention questionnaire was administered to both the intervention and control groups at the same time. Both groups completed a questionnaire containing items to measure knowledge, attitude, and self-efficacy for CPR. The investigator collected the completed questionnaires. The post-intervention questionnaire was administered to both the intervention and control groups at the same time, immediately after the end of the education program. The questionnaire was identical to the pre-intervention questionnaire, measuring participants’ knowledge, attitude, and self-efficacy for CPR. The investigator collected the completed questionnaires (see Additional file 1).

### Instruments

#### Knowledge

Knowledge was measured with an instrument developed by Byun [20] based on CPR guidelines published by the AHA in 2010. This 20-item scale comprised two items for checking for consciousness, two items for checking for breathing, seven items for delivering chest compressions, four items for maintaining airway and delivering rescue breaths, and five items for using a defibrillator. The total score ranged from 0 to 20, with a higher score indicating a higher level of knowledge.

#### Attitude

Attitude was measured using an instrument developed by Cho [21] with reference to the AHA guidelines and KACPR guidelines. Three types of attitudes were measured. Emotional attitude, which refers to one’s feelings about “performing basic CPR to a cardiac arrest patient,” was measured with 10 items rated on a seven-point scale. Behavioral attitude was measured with three items, including “I will try my best to perform CPR when I witness a cardiac arrest patient,” rated on a four-point scale. Finally, cognitive attitude was measured with three items, including “I think performing CPR promptly is important for the outcome of a cardiac arrest patient,” rated on a four-point scale. Five items for emotional attitude were reversely scored. The total score ranges from 0 to 94, with a higher score indicating a more positive attitude. The Cronbach’s α for emotional, behavioral, and cognitive attitudes in Cho’s [21] study were 0.69, 0.77, and 0.63, respectively. The Cronbach’s α for emotional attitude, behavioral attitude, and cognitive attitude in this study were 0.63, 0.85, and 0.87, respectively.

#### Self-efficacy

Self-efficacy was measured using a self-efficacy scale developed by Park [22] and modified and adapted by Byun [20]. The scale comprises 12 items, including “I am confident that I can perform CPR during an emergency.” The total score ranges from 0 to 120, with a higher score indicating a higher level of self-efficacy. The reliability (Cronbach’s α) of the tool in Park’s [22] study was 0.93, and that in this study was 0.90.

### Statistical analysis

The collected data were analyzed with the Statistical Package for the Social Sciences (SPSS) 22 software (IBM SPSS Statistics for Windows, Version 22.0. Armonk, NY: IBM Corp., 2013). Participants’ general characteristics were presented as a real number and percentage and as mean and standard deviation, and pre-intervention homogeneity between the groups was tested via χ^2^-test, *t*-test, and ANOVA. The changes after education in the intervention and control groups were analyzed with paired *t*-test, and differences for the intervention between the two groups were analyzed with ANCOVA with knowledge, which differed between the two groups, as the covariate.

## Results

### Study population

The general characteristics of the two groups are presented in Table 2. No significant differences in general characteristics were found between the intervention and control groups. In addition, two groups did not significantly differ in any of the outcome variables except for CPR knowledge.

**Table 2:** Homogeneity test of demographic characteristics and outcome variables in pre-test (*N* = 120)

Variables		Exp (*n* = 60)	Con (*n* = 60)	*t*	*p*
		*M* (*SD*)	*M* (*SD*)		
Age (years)		21.18 (1.08)	21.27 (1.16)	0.41	.685
Self-efficacy		71.51 (19.04)	69.31 (22.78)	−0.57	.567
Knowledge		7.98 (3.32)	6.63 (2.76)	−2.42	.017
Attitude	Emotion	37.70 (8.08)	35.90 (8.06)	−1.22	.225
	Behavior	7.03 (1.51)	7.56 (1.75)	1.78	.077
	Cognition	7.25 (1.49)	7.51 (1.76)	0.90	.373
		*n* (%)	*n* (%)	χ^2^	*p*
Gender	Female	50 (83.4%)	50 (83.4%)	0.001	.999
	Male	10 (16.6%)	10 (16.6%)		
Grades	1st	20 (33.3%)	20 (33.3%)	0.001	.999
	2nd	20 (33.3%)	20 (33.3%)		
	3rd	20 (33.3%)	20 (33.3%)		
Clinical practice	Yes	20 (33.3%)	20 (33.3%)	0.001	.999
	No	40 (66.7%)	40 (66.7%)		
Have you ever observed CPR?	Yes	18 (30%)	13 (21.6%)	1.09	.297
	No	42 (70%)	47 (78.4%)		
Have you ever had CPR training?	Yes	47 (78.3)	43 (71.7%)	0.71	.399
	No	13 (21.7%)	17 (28.3%)		
Have you ever done CPR?	Yes	11 (18.3%)	9 (15%)	0.24	.624
	No	49 (81.7%)	51 (85%)		

*Note. M* Mean, *SD* Standard Deviation, *CPR* Cardiopulmonary resuscitation, *Exp* Experimental Group, *Con* Control Group

### Hypothesis testing

#### Hypothesis 1

After receiving blended learning CPR education, the intervention group’s CPR knowledge score significantly increased from 7.98 (*SD =* 3.32) to 16.40 (*SD =* 1.56.) Thus, Hypothesis 1 was supported (Table 3).

**Table 3:** Mean changes in knowledge, attitude, and self-efficacy scores of participants (*N* = 120)

	Intervention group (*n* = 60)				Control group (*n* = 60)			
	*M* (*SD*)		*t*	*p*	*M* (*SD*)		*t*	*p*
	pre-test	post-test			pre-test	post-test		
Knowledge	7.98 (3.32)	16.40 (1.56)	−18.063	< .001	6.63 (2.76)	6.47 (2.63)	0.342	.734
Attitude								
Emotion	37.70 (8.09)	40.85 (8.01)	−2.563	.013	35.9 (8.07)	36.05 (6.87)	−0.110	.913
Behavior	7.03 (1.51)	7.60 (1.22)	−2.057	.044	7.57 (1.75)	7.63 (1.15)	− 0.256	.799
Cognition	7.25 (1.49)	7.93 (1.26)	−5.294	< .001	7.52 (1.76)	7.60 (1.48)	−0.897	.374
Self-efficacy	71.51 (19.04)	82.01 (18.39)	−3.632	.001	69.32 (22.78)	75.25 (16.38)	−1.731	.089

*Note. M* Mean, *SD* Standard Deviation

#### Hypothesis 2

After receiving blended learning CPR education, the intervention group’s CPR attitude scores significantly increased from 37.70 (*SD =* 8.09) to 40.85 (*SD =* 8.01) for emotion, from 7.03 (*SD =* 1.5) to 7.60 ((*SD =* 1.22) for behavior, and 7.25 (*SD =* 1.49) to 7.93 (*SD =* 1.26). Thus, Hypothesis 2 was supported (Table 3).

#### Hypothesis 3

After receiving blended learning CPR education, the intervention group’s CPR self-efficacy score significantly increased from 71.51 (*SD =* 19.04) to 82.01 (*SD =* 1 8.39.) Thus, Hypothesis 3 was supported (Table 3).

#### Hypothesis 4

The intervention group that received blended learning CPR education had a significantly higher CPR knowledge score 16.40 (*SD =* 1.56) than that of the control group 6.46 (*SD =* 2.62). Thus, Hypothesis 4 was supported (Table 4).

**Table 4:** Comparison of differences in scores between groups (*N* = 120)

Variables	Exp. (*n* = 60)	Con. (*n* = 60)	*F*	*p*	95% CI	η_p_^2^
	*M* (*SD*)	*M* (*SD*)				
Knowledge	16.40 (1.56)	6.46 (2.62)	595.78	< .001	9.10–10.71	.836
Attitude						
Emotion	40.85 (8.01)	36.05 (6.87)	9.61	.002	1.55–7.04	.076
Behavior	7.60 (1.22)	7.63 (1.15)	0.001	.979	−0.45-0.44	.000
Cognition	7.93 (1.26)	7.60 (1.48)	0.89	.348	−0.26-0.74	.008
Self-efficacy	82.02 (18.39)	75.25 (16.38)	3.44	.066	−0.41-12.49	.029

*Note. M* Mean, *SD* Standard Deviation*, Exp.* Experimental Group, *Con.* Control Group, *CI* Confidence Interval

#### Hypothesis 5

The intervention group that received blended learning CPR education had a significantly higher CPR emotional attitude score 40.85 (*SD =* 8.01) than that of the control group 36.05 (*SD =* 6.87), with no significant differences in other two components of attitude. Thus, Hypothesis 5 was partially supported (Table 4).

#### Hypothesis 6

The intervention group that received blended learning CPR education had a higher CPR self-efficacy score 82.02 (*SD =* 18.39) than that of the control group 75.25 (*SD =* 16.38), but the difference was not statistically significant. Thus, Hypothesis 6 was not supported (Table 4).

## Discussion

By analyzing the effects of a blended learning program based on e-learning materials developed by major Korean public health institutions with government funding on CPR knowledge, attitude, and self-efficacy, this study has added new evidence supporting the effectiveness of CPR blended learning. The most notable accomplishment was that the intervention group that underwent this blended learning program showed significant improvements in their scores in knowledge, emotional attitude, behavioral attitude, cognitive attitude, and self-efficacy following the completion of the e-learning program. In this study, the intervention group underwent CPR education via blended learning. They had a higher CPR knowledge score after education than the control group. This aligns with the findings of a previous study that analyzed the effects of a web course called “Help-brain-heart,” before CPR training on knowledge, CPR skills, and willingness to act in teenagers [23], where web-based learning improved students’ theoretical knowledge of acute myocardial infarction (AMI) and stroke and lifestyle factors. Furthermore, our findings align with those of a Korean study on nurses who received basic CPR education using videos [24], where video-based education was effective in increasing knowledge in the single intervention group. This suggests that blended learning programs can improve learners’ knowledge scores. In other words, administering blended learning CPR education to nursing students seems desirable for increasing their knowledge of CPR.

Furthermore, the intervention group, which underwent CPR blended learning, had a significantly more positive emotional attitude toward CPR after education compared to that of the control group. This is identical to the finding of a Korean study that administered basic CPR education on Korean nurses using a video program [24], where the intervention group had significantly better belief and emotion scores of CPR attitudes after education. In other words, as with knowledge, the emotional aspect of attitude mostly improves after education. However, behavioral attitude was slightly lower, though insignificant, and cognitive attitude score was higher in the intervention group, but not to a significant extent. These findings support a previous report that because CPR is a psychomotor skill, hands-on clinical training is more effective than lecture-based or video-based education in improving CPR performance [25–28]. Some previous studies have confirmed the effectiveness of hands-on education in improving CPR performance [25, 26, 28]. However, a supplemental learning method that increases temporal and spatial flexibility of learning administered before and after hands-on training, to prepare participants for the training and reinforce their learning, would be a cost-effective method [29]. Moreover, such methods could appeal to younger generations and be used for rehearsals [28].

Following the e-Learning, the intervention group showed toward higher scores on the behavioral and cognitive aspects of attitude than did the control group. This aligns with the findings of a previous study that administered basic video-based CPR education to Korean nurses [24], where the intervention group did not show a significant difference in behavioral attitudes toward CPR after education. Furthermore, these results support a previous report that web lecture prior to CPR training does not affect practical CPR skills or willingness to act in nursing students [23]. Nevertheless, it was beneficial for increasing nursing students’ knowledge [23].

With clinical learning at its core, nursing education aims to help students learn and practice nursing skills and develop self-efficacy [30], and thus, confirming the importance of self-efficacy for the development of psychomotor skills. In this study, the intervention group showed significantly higher self-efficacy after education, and the intervention group’s self-efficacy score was higher than that of the control group, but not to a significant extent. This suggests that CPR education using a blended learning method is effective in improving students’ self-efficacy regarding the ability to perform CPR. Students’ self-efficacy refers to the scope or power of one’s beliefs about one’s ability to complete a task and accomplish the goal. Self-efficacy in nursing students prevents stress and burnout and increases learning performance by promoting participation in nursing education [30]. At the same time, self-efficacy is a predictor of academic success and personal development, as an increase of self-efficacy promotes one’s participation in learning, and thereby improves learning outcomes [30].

Overall, CPR education using a video titled “How to perform chest compression CPR and use automated defibrillator” produced by the KACPR was effective in increasing knowledge and emotional attitude but not behavioral and cognitive attitude nor self-efficacy compared to a control group. Therefore, it would be beneficial to use CPR blended learning programs that integrate lectures and videos to educate nursing students in CPR. Although this study has demonstrated the effectiveness of blend learning in CPR education, some studies suggest that there are shortcomings in education using video [31, 32]. Thus, when designing blended learning of CPR education, efforts will be needed to compensate for these burdensome.

One limitation of this study is that the participants were nursing students studying in the same school, so the possibility of diffusion could not be completely eliminated. Furthermore, generalization of the findings is limited, as only nursing students from one school were examined. Second, the pre-knowledge levels for CPR in the two groups differed in this study. It should be interpreted with caution that the findings that the differences were adjusted by statistical techniques but differed in the knowledge of the CPRs between the control and intervention groups. Finally, this study could not directly assess CPR psychomotor skill performance. Nor could this study provide training or assessment in CPR performance, due to the nature of the study procedure.

## Conclusions

This study, which investigated the effects of a CPR blended learning program, confirmed that integrating existing CPR instruction videos was effective in increasing CPR knowledge and emotional attitude but not behavioral and cognitive attitude nor self-efficacy compared to control group. In today’s clinical practice, there is a high demand for well-trained nursing graduates who are capable of effectively responding to various service needs in complicated environments. Shifting nursing education to one that integrates traditional education with skill training would minimize the gap between service deliveries, enhance the quality of education, and ensure the safety of patients.

## Supplementary information

**Additional file 1.** The Supplementary data for nursing students’ knowledge, attitude, self-efficacy in blended learning of cardiopulmonary resuscitation: a randomized controlled trial. Pre/post-intervention questionnaires.

## Data Availability

The datasets used and/or analyzed during the current study are available from the corresponding author on reasonable request.

## Abbreviations

AHA: American Heart Association
AMI: Acute myocardial infarction
CPR: Cardiopulmonary resuscitation
CVDs: Cardiovascular diseases
KACPR: Korean Association of Cardiopulmonary Resuscitation
KCDC: Korea Centers for Disease Control and Prevention
MOHW: Ministry of Health and Welfare
OECD: Organisation for Economic Co-operation and Development
RCTs: Randomized controlled trials
SD: Standard deviation
SPSS: Statistical Package for the Social Sciences
