# Supplementary material for: Nursing students’ knowledge, attitude, self-efficacy in blended learning of cardiopulmonary resuscitation: a randomized controlled trial
Source: BMC Med Educ. 2019 Nov 9;19:414. doi: 10.1186/s12909-019-1848-8 (PMC6842519; doi:10.1186/s12909-019-1848-8)
Supplement: Supplementary file 1 — Additional file 1. The Supplementary data for nursing students’ knowledge, attitude, self-efficacy in blended learning of cardiopulmonary resuscitation: a randomized controlled trial. Pre/post-intervention questionnaires. [file 12909_2019_1848_MOESM1_ESM.docx]

**Supplementary data**

**For**

**Nursing Students’ Knowledge, Attitude, Self-Efficacy in Blended E-Learning of Cardiopulmonary Resuscitation:** **a randomized controlled trial**

The actual questionnaire was presented, written, and completed in Korean.

**Pre/post-intervention questionnaires**

**1. Knowledge**

The following items are about CPR knowledge. Please read each question and make a "X" mark where the content matches your thinking.

| Domain | Questions | Score | | |
| --- | --- | --- | --- | --- |
|  |  | True  (1) | False  (0) | Don’t know  (0) |
| Confirm the reaction  (1) | Simultaneously, confirm the presence of consciousness and breathing. |  |  |  |
| Request help  (1) | If you witness a fallen adult patient with cardiac arrest, call 119 after performing a cardiopulmonary resuscitation for two minutes. |  |  |  |
| Circular request  (2) | The pulse palpation area of pediatric cardiac arrest patients is the brachial artery. |  |  |  |
|  | The pulse verification time is 5-10 seconds. |  |  |  |
| Chest compression  (7) | The order of CPR is Circulation - Airway - Breathing. |  |  |  |
|  | The chest compression position is the upper half of the sternum. |  |  |  |
|  | The chest compression rate is 100-120 cycles per minute for both adults and infants. |  |  |  |
|  | Chest compression depth is 5-6 cm for adults. |  |  |  |
|  | In adult cardiac arrest patients, the ratio of chest compression to artificial respiration during rescuer CPR is 15: 2. |  |  |  |
|  | A good way to relieve the pressure on the chest is to remove the weight of the rescuer who has been on the chest. |  |  |  |
|  | If you are a non-medical person and you find an adult cardiac arrest patient, you can do CPR with only chest pressure. |  |  |  |
| Airway maintenance and artificial respiration  (4) | For patients with cardiac arrest with signs of trauma, open the airway by head tilt-chin lift. |  |  |  |
|  | If artificial respiration does not result in breathing after two attempts at artificial respirations, stop breathing and immediately perform chest compressions. |  |  |  |
|  | Artificial respiration is performed every 3-5 seconds for an adult with a pulse and no breathing. |  |  |  |
|  | Even if the tracheal tube is intubated, the ratio of chest compressions to artificial respiration remains at 30: 2. |  |  |  |
| Defibrillation  (5) | For adults, pediatric automatic defibrillators may be used. |  |  |  |
|  | If one rescuer is performing adult cardiopulmonary resuscitation independently, the automatic defibrillator is activated immediately when the defibrillator arrives. |  |  |  |
|  | When the automatic defibrillator is used for heart rhythm analysis, chest compressions may be performed. |  |  |  |
|  | Chest compressions are performed while the automatic defibrillator is being charged. |  |  |  |
|  | Immediately after defibrillation with automatic defibrillator (AED), chest compressions are performed. |  |  |  |

**2. Emotional attitudes**

The following is a list of words that describe feelings or emotions about performing cardiopulmonary resuscitation in an adult cardiac arrest patient within the hospital. For each word, please indicate "X" in the place that best matches your feelings or emotion.

“It is _________ for me to perform basic cardiopulmonary resuscitation to patients with cardiac arrest.”

|  | Very | Generally | Slightly | Usually | Slightly | Generally | Very |  |
| --- | --- | --- | --- | --- | --- | --- | --- | --- |
| Good |  |  |  |  |  |  |  | Bad |
| Beneficial |  |  |  |  |  |  |  | Harmful |
| Unnecessary |  |  |  |  |  |  |  | Necessary |
| Not Useful |  |  |  |  |  |  |  | Useful |
| Important |  |  |  |  |  |  |  | Not Important |
| Not Afraid |  |  |  |  |  |  |  | Afraid |
| Tense |  |  |  |  |  |  |  | Not Tense |
| Uneasy |  |  |  |  |  |  |  | Not Uncomfortable |
| Not Frustrated |  |  |  |  |  |  |  | Frustrated |
| Stressful |  |  |  |  |  |  |  | Not Stressful |

**3. Behavioral attitudes**

Read the following questions related to cardiopulmonary resuscitation in a cardiac arrest patient in the hospital and make a "X" mark in line with your opinion.

|  | Really it is | Usually it is | Usually not | Not very |
| --- | --- | --- | --- | --- |
| 1. Cardiopulmonary resuscitation for patients experiencing cardiac arrest is an important part of nursing practice. |  |  |  |  |
| 2. I will do my best to perform CPR when I actually witness a patient with cardiac arrest. |  |  |  |  |
| 3. I plan to actively resume cardiopulmonary resuscitation in advance of other medical staff or fellow nurses when I witness a patient having an actual cardiac arrest. |  |  |  |  |

**4. Cognitive attitudes**

In the hospital, read the following questions related to cardiopulmonary resuscitation in a cardiac arrest patient and make a "X" mark in line with your opinion.

|  | Really it is | Usually it is | Usually not | Not very |
| --- | --- | --- | --- | --- |
| 1. When a cardiac arrest occurs, I think I should go ahead and perform CPR. |  |  |  |  |
| 2. I believe that rapid cardiopulmonary resuscitation is important for the prognosis of cardiac arrest patients. |  |  |  |  |
| 3. I think my role has a direct impact on the outcome of CPR. |  |  |  |  |

**5. Self-efficacy**

This is a statement regarding how well you think you can do the following things. Please mark "○" in the place that best expresses your confidence level. Please indicate your current state, not your desired state.

|  | I'm not sure at all I am very confident | | | | | | | | | | |
| --- | --- | --- | --- | --- | --- | --- | --- | --- | --- | --- | --- |
| 1. I am confident that I can perform cardiopulmonary resuscitation in an emergency. | 0 | 1 | 2 | 3 | 4 | 5 | 6 | 7 | 8 | 9 | 10 |
| 2. I have not been able to learn CPR because it is too difficult to perform CPR. | 0 | 1 | 2 | 3 | 4 | 5 | 6 | 7 | 8 | 9 | 10 |
| 3. I do not seem to be able to perform CPR in an emergency situation. | 0 | 1 | 2 | 3 | 4 | 5 | 6 | 7 | 8 | 9 | 10 |
| 4. I believe in my own CPR abilities. | 0 | 1 | 2 | 3 | 4 | 5 | 6 | 7 | 8 | 9 | 10 |
| 5. I can accurately identify the nature of the patient's emergency. | 0 | 1 | 2 | 3 | 4 | 5 | 6 | 7 | 8 | 9 | 10 |
| 6. I can quickly respond to a patient's emergency. | 0 | 1 | 2 | 3 | 4 | 5 | 6 | 7 | 8 | 9 | 10 |
| 7. I can confirm if a patient is conscious or unconscious. | 0 | 1 | 2 | 3 | 4 | 5 | 6 | 7 | 8 | 9 | 10 |
| 8. I can call 119 when I encounter an unconscious patient. | 0 | 1 | 2 | 3 | 4 | 5 | 6 | 7 | 8 | 9 | 10 |
| 9. I can confirm the breathing of unconscious patients. | 0 | 1 | 2 | 3 | 4 | 5 | 6 | 7 | 8 | 9 | 10 |
| 10. I perform artificial respiration to a patient who is not breathing. | 0 | 1 | 2 | 3 | 4 | 5 | 6 | 7 | 8 | 9 | 10 |
| 11. I can identify the carotid arteries in an unconscious patient. | 0 | 1 | 2 | 3 | 4 | 5 | 6 | 7 | 8 | 9 | 10 |
| 12. I can perform artificial respiration and chest compressions on a patient who has no pulse. | 0 | 1 | 2 | 3 | 4 | 5 | 6 | 7 | 8 | 9 | 10 |
